# Long Noncoding RNA MIR210HG Promotes the Warburg Effect and Tumor Growth by Enhancing HIF-1α Translation in Triple-Negative Breast Cancer

**DOI:** 10.3389/fonc.2020.580176

**Published:** 2020-12-17

**Authors:** Ye Du, Na Wei, Ruolin Ma, Shu-Heng Jiang, Dong Song

**Affiliations:** ^1^Departments of Breast Surgery, The First Hospital of Jilin University, Changchun, China; ^2^State Key Laboratory of Oncogenes and Related Genes, Shanghai Cancer Institute, Ren Ji Hospital, School of Medicine, Shanghai Jiao Tong University, Shanghai, China

**Keywords:** long noncoding RNA, triple-negative breast cancer, Warburg effect, MIR210HG, HIF-1α

## Abstract

**Background:**

Hypoxia is an important environmental factor and has been correlated with tumor progression, treatment resistance and poor prognosis in many solid tumors, including triple-negative breast cancer (TNBC). Emerging evidence suggests that long noncoding RNA (lncRNA) functions as a critical regulator in tumor biology. However, little is known about the link between hypoxia and lncRNAs in TNBC.

**Methods:**

TNBC molecular profiles from The Cancer Genome Atlas (TCGA) were leveraged to identify hypoxia-related molecular alterations. Loss-of-function studies were performed to determine the regulatory role of MIR210HG in tumor glycolysis. The potential functions and mechanisms of hypoxia-MIR210HG axis were explored using qPCR, Western blotting, luciferase reporter assay, and polysome profiling.

**Results:**

We found that MIR210HG is a hypoxia-induced lncRNA in TNBC. Loss-of-function studies revealed that MIR210HG promoted the Warburg effect as demonstrated by glucose uptake, lactate production and expression of glycolytic components. Mechanistically, MIR210HG potentiated the metabolic transcription factor hypoxia-inducible factor 1α (HIF-1α) translation via directly binding to the 5’-UTR of HIF-1α mRNA, leading to increased HIF-1a protein level, thereby upregulating expression of glycolytic enzymes. MIR210HG knockdown in TNBC cells reduced their glycolytic metabolism and abolished their tumorigenic potential, indicating the glycolysis-dependent oncogenic activity of MIR210HG in TNBC. Moreover, MIR210HG was highly expressed in breast cancer and predicted poor clinical outcome.

**Conclusion:**

Our results decipher a positive feedback loop between hypoxia and MIR210HG that drive the Warburg effect and suggest that MIR210HG may be a good prognostic marker and therapeutic target for TNBC patients.

## Background

Triple-negative breast cancer (TNBC) a specific subtype of breast cancer that does not express progesterone receptor, estrogen receptor, and human epidermal growth factor receptor 2 (HER2, overexpression and/or amplification). TNBC constitutes ~15% of all breast cancer subtypes and exhibits high invasiveness, increased metastatic potential, high risk of recurrences, and poor outcomes (1). Due to the limited targeted therapy available for this deadly disease, the clinical treatment of TNBC is still hindered by metastasis or recurrence (2, 3). Therefore, a more comprehensive understanding of the molecular mechanisms that implicated in TNBC progression will likely aid in clinical developmental therapeutics.

A common feature of many solid tumors is hypoxia due to insufficient blood supply (4). Hypoxia-inducible factor 1 alpha (HIF-1α), functioning as first responder under hypoxic conditions, is highly expressed in TNBC. Hypoxia leads to HIF-1α stabilization and rapid protein accumulation and increased transcriptional activity (5). Through transcription of several hundred of target genes, HIF-1α is profoundly implicated in many malignant phenotypes, such as angiogenesis, metabolic reprogramming, stemness maintenance, cell survival and proliferation, tumor motility and invasion, immune evasion, and resistance to chemoresistance (6–9). Recent preclinical studies showed that the combination of cytotoxic chemotherapy with drugs that targeting hypoxia-inducible factors may improve the clinical outcome for TNBC patients (10–12). 

Long non-coding RNAs (lncRNAs) are a class of nonprotein-coding RNAs with a length of more than 200 nucleotides (13). Accumulating evidence has shown that lncRNAs are participated in many fundamental cellular functions, such as histone modification, alternative splicing, chromatin structure modification, and gene expression regulation (14). Recently, lncRNAs have emerged as crucial regulators of cell differentiation, organogenesis, and tumorigenesis (15, 16). In TNBC, dysregulation of many lncRNAs has been reported to promote cell survival, tumor metastasis, immune evasion, and chemoresistance (17–20). For example, lncRNA LINK-A expression in TNBC cells contributes to downregulation of antigenicity and facilitates intrinsic tumor suppression (21). Moreover, lncRNA NRAD1 regulates expression of genes involved in differentiation and catabolic processes, which are essential for TNBC development (22).

Reprogrammed energy metabolism is an emerging hallmark of human cancers (23). Highly proliferative cancer cells preferentially metabolize glucose by aerobic glycolysis rather than through the more energetically efficient oxidative phosphorylation, even in the presence of sufficient oxygen, a phenomenon known as the Warburg effect (24, 25). Accumulating evidence suggests that the Warburg effect is closely associated with a poor clinical outcome and exerts critical implications on tumor progression (26, 27). In this study, we first performed integrated analysis to characterize hypoxia-related lncRNAs in TNBC through leveraging large-scale TNBC molecular profiles from The Cancer Genome Atlas (TCGA). Subsequently, functional verification showed that MIR210HG is a hypoxia-induced lncRNA and acts as a key glycolytic regulator in TNBC. Importantly, MIR210HG regulates glycolytic gene expression through increased HIF-1α mRNA translation. Therefore, our data revealed a novel feedback loop between HIF-1α and MIR210HG that facilitates the Warburg effect in TNBC, suggesting that targeting HIF-1α/MIR210HG axis could be a potential therapeutic target.

## Materials and Methods

### Bioinformatics Analysis

The RNA-sequencing data of TNBC and corresponding non-tumor tissues were downloaded from The Cancer Genome Atlas (TCGA, https://gdc.cancer.gov/) database. A well-documented 15-gene expression signature (ACOT7, ADM, ALDOA, CDKN3, ENO1, LDHA, MIF, MRPS17, NDRG1, P4HA1, PGAM1, SLC2A1, TPI1, TUBB6, and VEGFA) was used to classify hypoxia status (11). These genes are mainly targets of HIF1A. As reported previously, this gene signature was derived by selecting genes that were consistently co-expressed with the hypoxia seeds in multiple cancers and defined based on gene function and analysis of *in vivo* co-expression patterns (28). Differentially expressed lncRNAs related to hypoxia status were analyzed by estimating an exact test P-value.

### Cell Lines

Human breast cancer cell lines (MCF7, T47D, ZR-75-1, Hs578T, MDA-MB-231, and HCC1937) and the non-malignant human mammary epithelial cell lines MCF10A were all obtained from Cell Resource Center of Shanghai Institutes for Biological Sciences, Chinese Academy of Sciences (Shanghai, China). Cells were cultured in RPMI-1640 (Hyclone, USA) or Dulbecco’s modified Eagle’s medium (Hyclone, USA) supplemented with 10% fetal bovine serum (Gibco, USA), 100 U/mL penicillin, and 100 μg/mL streptomycin (Invitrogen, USA). All cell lines were cultured in a humidified incubator of 5% CO_2_ at 37°C.

### Small Interfering RNA and Generation of Stably Expressing Cell Lines

For siRNA transfection, Hs578T, MDA-MB-231, and HCC1937 cells were seeded in six-well plates and allowed to grow to 50–70% confluence. Then, the cells were transfected with HIF-1α siRNA or negative control (GenePharma Inc., Shanghai, China) at a concentration of 50 nM using Lipofectamine 2000 reagent (Invitrogen, USA) according to the manufacturer’s instructions. To generate stable MIR210HG-depleted TNBC cells, the pCDH-CMV-MCS-EF1-copRFP lentiviral vector was used. The shRNA sequences were shown as follows: sh-MIR210HG-1, 5′-GCATTAGTACAGGCACCAGCCTA-3′; sh-MIR210HG-2, 5′-UUUAGACCCAUUCUCGUAUGGAGGU-3′. A non-silencing shRNA (sh-Ctrl) oligonucleotide was used as a negative control.

### Real-Time Quantitative PCR

Total RNAs from breast cancer cells or tumor tissues were extracted by the RNAiso Plus kit (Takara Bio Inc., Japan). Twenty-two TNBC specimens were also obtained from Departments of Breast Surgery, The First Hospital of Jilin University. All the patients were provided with written informed consent before enrollment, and the study was approved by the Research Ethics Committee of The First Hospital of Jilin University. The RNA concentration and quality were determined by spectrophotometry using NanoDrop™ 2000 (Thermo Scientific, USA). Then, RNA was reverse transcribed to complementary DNA (cDNA) by using a PrimeScript^TM^ 1^st^ Strand cDNA Synthesis Kit (Takara Bio, Shiga, Japan) according the manufacturer’s instructions. Quantitative real-time PCR was performed with SYBR Green using the ViiA7 System (AB Applied Biosystems, USA). ACTB mRNA was used for normalization. The primers used in this study were shown in **Supplementary Table 1**.

### Isolation of Cytoplasmic and Nuclear RNA

Nuclear and cytoplasmic fractions were isolated by the PARIS Kit (Life Technologies, USA) according to the manufacturer’s instruction. GAPDH and U1 were used as cytoplasmic and nuclear controls, respectively. Then, isolated RNA was subjected to reverse transcription reaction and real-time qPCR. The primers used were shown as follows: GAPDH, forward: 5’-CTGGGCTACACTGAGCACC-3’, reverse: 5’-AAGTGGTCGTTGAGGGCAATG-3’; U1, forward: 5’-GTGGTTTTTCCAGAGCAAGG-3’, reverse: 5’-CAGGGGAAAACACAGACACA-3’.

### Western Blotting

Cells were lysed with lysis buffer containing 0.1% Triton X-100, 20 mM Tris-Cl, 125 mM NaCl, 0.5 mM EDTA, 1 mM dithiothreitol (DTT), and protease inhibitor cocktail. Protein concentration was detected by Pierce BCA Protein assay kit (Thermo Fisher Scientific, USA). Lysates were separated by sodium dodecyl sulfate (SDS)-polyacrylamide gel electrophoresis, and transferred onto polyvinylidene fluoride (PVDF) membranes (Millipore, USA). The membrane was then blocked with 5% non-fat milk and hybridized overnight with primary antibodies against HIF-1α (#36169, Cell Signaling Technology), GLUT1 (21829-1-AP, ProteinTech), PKM2 (15822-1-AP, ProteinTech), LDHA (19987-1-AP, ProteinTech), and β-actin (ab8227, Abcam). The next day, blots were detected with horseradish peroxidase-conjugated anti-IgG for 1 h at room temperature and visualized with an ECL kit (Millipore, USA).

### Measurement of Glucose and Lactate Level

Glucose utilization and lactate release by cancer cells were used to detect cellular glycolytic activity as reported previously (29). Briefly, 1 × 10^6^ indicated cells were seeded in 60-mm plates and supplemented with FBS-free medium. After incubation for 24 h, culture medium was collected and subjected for glucose and lactate level analysis using a commercial glucose assay kit (Sigma-Aldrich, MAK263, Shanghai, China) and a Lactate Assay Kit (BioVision, K607-100, USA) according to the manufacturer’s instruction. Total cell protein was used for normalization.

### Polysome Profiling

Polysome profiling is a method widely used to monitor the translation activity of mRNAs. Once each polysome fractions are collected, the translation activity of each mRNA can be analyzed using various molecular biology techniques such as Northern blotting and RT-PCR. Polysome profiling was performed as reported elsewhere (30). Briefly, cell lysates were collected with polysome lysis buffer and then loaded onto 10 to 50% sucrose density gradients prepared in polysome buffer. After centrifugation for 3 h at 35,000 rpm at 4°C, gradients were recovered in 12 fractions using gradient fractionators and RNA was isolated from each fraction. The expression of HIF-1α mRNA was detected by quantitative reverse transcription PCR.

### Immunofluorescence

RNA-FISH was performed with MIR210HG specific probe designed and synthesized by ServiceBio Company (Wuhan, China). In brief, MDA-MB-231 and HCC1937 cells were fixed with 4% paraformaldehyde for 10 min. Then, the cells were permeabilized by 0.5% TritonX-100 for 5 min at 4°C and washed with PBS for three times. Hybridization was performed with MIR210HG probe in a moist chamber at 37°C overnight, flowing by co-staining with DAPI for 10 min. After that, the cells were washed and photographed with a fluorescence confocal microscope.

### Immunohistochemistry (IHC)

IHC was performed on formalin-fixed paraffin-embedded sections as reported previously (31). After deparaffinization and citrate-based antigen retrieval, endogenous peroxidase was blocked by 3% H_2_O_2_. Then, the sections were washed and incubated with primary antibodies against Ki67 (#9027, Cell Signaling Technology) or HIF-1α (#36169, Cell Signaling Technology) at 4°C overnight. The next day, slides were incubated with a second antibody labeled by HRP at room temperature for 1 h. Finally, slides were developed with the HRP substrate diaminobenzidine and counterstained with hematoxylin. Scoring was mainly conducted based on the percentage of positive-staining cells: 0–5% scored 0, 6–30% scored 1, 30–70% scored 2, and more than 70% scored 3. The final score was designated as low or high expression as follows: low expression (score 0–1), high expression (score 2–3). These scores were determined independently by two senior pathologists in a blinded manner.

### Chromatin Immunoprecipitation Assay

Chromatin immunoprecipitation (ChIP) experiment was performed using the ChIP assay kit (Pierce Agarose ChIP Kit). In brief, HS578T, MDA-MB-231, and HCC1937 cells were crosslinked and sonicated, and DNA was immunoprecipitated with HIF-1α (#36169, Cell Signaling Technology) or isotype-matched control IgG (Cell Signaling Technology) from the sonicated cell lysates and quantified using Premix Taq™ PCR analysis (Takara, Japan). The primers used in the assay were shown as follows. Site 1: forward, 5’-CCCGGGCAGACGTGC-3’; reverse, 5’-CCTGGTCCCTCAGCCAATG-3’; Site 2: forward, 5’-GTCACGGCCCGGGATAC-3’; reverse, 5’-GGAGCTGCCCCTCTTCCC-3’. The known target of HIF-1α vascular endothelial growth factor A (VEGFA) was used as a positive control.

### uciferase Reporter Assay

Two hundred ninety-two base pairs of human HIF-1α, 5’-untranslated region (UTR) upstream of the start codon (identified by UCSC Genome Browser, http://www.genome.ucsc.edu/), were amplified and cloned into bicistronic reporter plasmid. MDA-MB-231 and HCC1937 cells were seeded in 6-well plates at a density of 2 × 10^5^ cells per well. After incubation overnight, the reporter was transfected into stable sh-MIR210HG or sh-Ctrl MDA-MB-231 and HCC1937 cells for 48 h. Finally, the cells were harvested and luciferase activity was analyzed using Dual-Glo Luciferase Assay (Promega). Renilla activity was used to an internal control. Moreover, mRNA expression of firefly-luciferase was measured as an additional control.

### Measurement of HIF-1α Transcription Activity

Briefly, a specific double stranded DNA sequence containing the HIF-1α response element (5’-ACGTG-3’) is immobilized to the wells of a 96-well plate. The nuclear extract lysates from sh-Ctrl and sh-MIR210HG MDA-MB-231 and HCC1937 cells were harvested using the Nuclear Extraction kit (ab113474; Abcam, Cambridge, UK). Then, HIF-1α activity was detected by addition of a specific primary antibody directed against HIF-1α according to the manufacturer’s instruction (ab133104; Abcam, Cambridge, UK). A secondary antibody conjugated to HRP is added to provide a sensitive colorimetric readout at 450 nm.

### CCK-8 Assay

For CCK-8 experiment, MDA-MB-231 and HCC1937 cells were seeded in 96-well plates at 3,000 cells per well. At indicated time points (day 1, 3, 5), cell viability was measured by treatment with 10% (v/v) CCK-8 solution (Dojindo, Kumamoto, Japan) for 1 h at 37°C. Then, the absorbance value at 450 nm in each well was detected by a microplate reader.

### Animal Experiment

BALB/c nude male mice aged 6 weeks were obtained from Shanghai Jiesijie Laboratory Animal Co., Ltd. Mice were manipulated and housed according to the criteria outlined in the “Guide for the Care and Use of Laboratory Animals” prepared by the National Academy of Sciences. Mice were kept on a 12-hour day/night cycle with free access to food and water. Mice were divided into two groups at random. The investigator was blinded to the group allocation during the experiment. For generation of subcutaneous xenograft model, mice were subcutaneously injected with 1 × 10^6^ sh-control or sh-MIR210HG-depleted MDA-MB-231 cells under the right lower limbs, followed by growth under specific pathogen-free condition for 5 weeks. At the end of the experiment, all mice were euthanized and the tumors were resected, weighed and collected for IHC and real-time qPCR analysis. This study was approved by the Research Ethics Committee of The First Hospital of Jilin University.

### Statistical Analysis

Data were presented as the means ± SD. The statistical analysis was performed with GraphPad Prism 5 (GraphPad Software, San Diego, CA, USA). The two-sided Student’s t test or one-way ANOVA followed by Student-Newman-Keuls (SNK) test was used to compare data between groups. Survival time was calculated by the Kaplan-Meier method and analyzed by the log-rank test. Correlation analysis was evaluated by Spearman’s rank correlation. For all tests, a p-value of less than 0.05 was considered statistically significant.

## Results

### Integrated Analysis of Hypoxia-Related lncRNAs in TNBC

**Figure 1:**
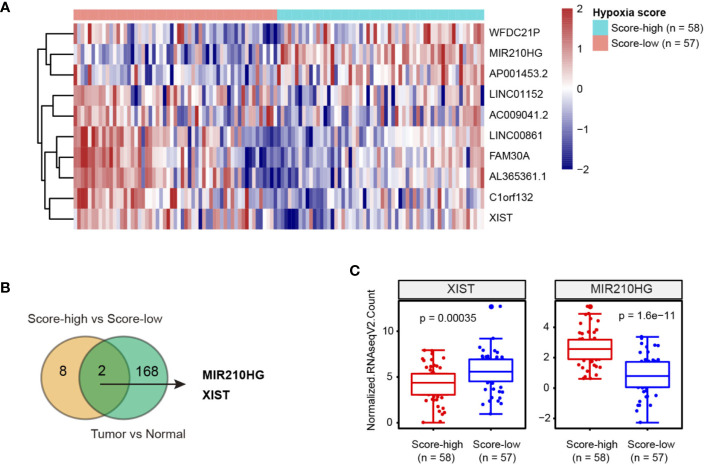
Integrated analysis of hypoxia-related lncRNAs in TNBC. **(A)** Heatmap of 10 lncRNAs related to hypoxia in TNBC; hypoxia score-low: n = 57 and hypoxia score-high: n = 58. **(B)** Venn diagram showed that 2 hypoxia-related lncRNAs were dysregulated in TNBC tissues. **(C)** Expression level of XIST and MIR210HG in hypoxia score-high (n = 58) and score-low (n = 57) groups.

RNA sequencing data of TNBC patient samples was acquired from The Cancer Genome Atlas (TCGA). To classify the hypoxia status of TNBC samples, we focused on a 15-gene expression signature (ACOT7, ADM, ALDOA, CDKN3, ENO1, LDHA, MIF, MRPS17, NDRG1, P4HA1, PGAM1, SLC2A1, TPI1, TUBB6, and VEGFA) that was well documented in a recent article assessing hypoxia-associated molecular features (11). Then, TNBC samples were stratified into hypoxia score-low (n = 57) or score-high (n = 58) groups (**Supplementary Table 2**). By comparing the differentially expressed lncRNAs, 10 lncRNAs (XIST, LINC01152, AL365361.1, C1orf132, FAM30A, AC009041.2, LINC00861, AP001453.2, WFDC21P and MIR210HG) had at least two-fold change (**Figure 1A**). Moreover, we revealed that 160 lncRNAs were differentially expressed in TNBC tissues compared with normal breast tissues (**Supplementary Figure 1**). Of note, 2 hypoxia-related lncRNAs (MIR210HG and XIST) were dysregulated (**Figure 1B**); MIR210HG expression was upregulated approximately 5 times in glycolysis-high TNBC tissues compared with glycolysis-low TNBC tissues (**Figure 1C**).

### MIR210HG Is Induced by Hypoxia in TNBC Cells

**Figure 2:**
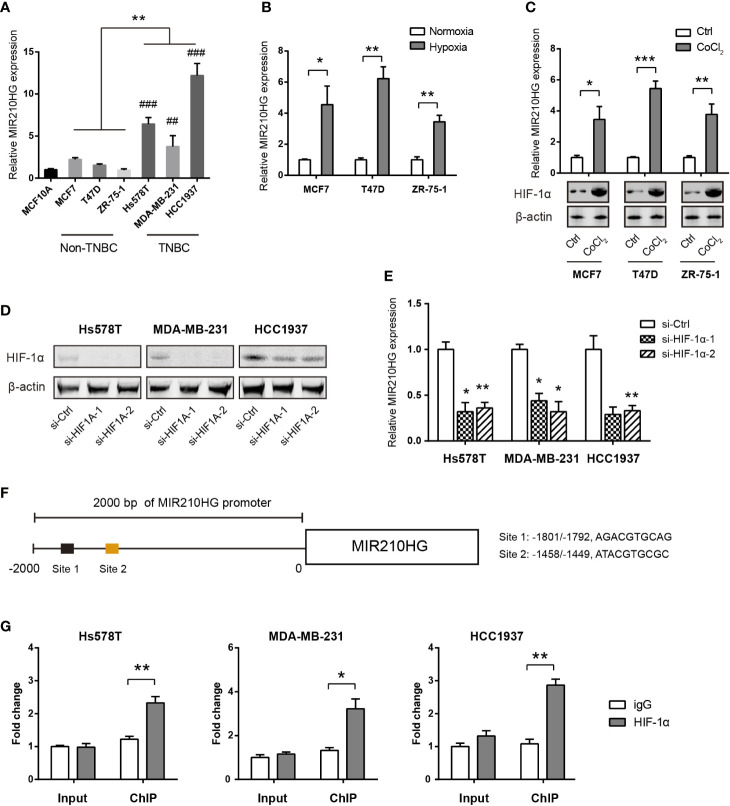
MIR210HG is induced by hypoxia in TNBC cells. **(A)** Real-time qPCR analysis of MIR210HG expression in breast cancer cells and the non-malignant MCF10A cells; indicates statistical significance of comparison to normal MCF10A cells; ^##^p < 0.01; ^###^p < 0.001. **(B)** Real-time qPCR analysis of MIR210HG expression in three breast cancer cell lines (MCF7, T47D, and ZR-75-1) under hypoxia (1% O_2_) or normoxia (20% O_2_) condition. **(C)** Real-time qPCR analysis of MIR210HG expression in three breast cancer cell lines (MCF7, T47D, and ZR-75-1) after treatment with CoCl_2_ (50 μM) for 24 h. **(D)** Western blotting analysis of HIF-1α knockdown efficiency in Hs578T, MDA-MB-231, and HCC1937 cells. **(E)** Real-time qPCR analysis of MIR210HG expression in three breast cancer cell lines (MCF7, T47D, and ZR-75-1) after HIF-1α knockdown. **(F)** A schematic diagram showed the HIF-1α locus in the MIR210HG promoter. **(G)** HIF-1α occupation on MIR210HG promoter was evaluated by ChIP-qPCR, IgG was used as negative control (n= 3). *p < 0.05; **p < 0.01; ***p < 0.001.

By real-time qPCR analysis, we found that MIR210HG expression was highly expressed in TNBC cells (Hs578T, MDA-MB-231 and HCC1937) compared to non-TNBC cells (MCF7, T47D, and ZR-75-1) and the non-malignant breast epithelial MCF-10A cells (**Figure 2A**). To test whether hypoxia is responsible for MIR210HG expression, we cultured MCF7, T47D and ZR-75-1 cells under normoxia or hypoxia condition for 24 h. The result showed that MIR210HG expression was differentially increased by hypoxia in these cell lines (**Figure 2B**). To mimic hypoxia, we treated MCF7, T47D and ZR-75-1 cells with 100 μM CoCl_2_, a known chemical inducer of HIF-1α. Expectedly, CoCl_2_ also significantly promoted MIR210HG expression (**Figure 2C**). Next, we genetically silenced HIF-1α in three TNBC cell lines. Two specific siRNAs against HIF-1α led to significant reduction in HIF-1α protein level (**Figure 2D**). As a result, HIF-1α knockdown remarkably suppressed MIR210HG expression in Hs578T, MDA-MB-231 and HCC1937 cells (**Figure 2E**). Moreover, chromatin immunoprecipitation experiment demonstrated that HIF-1α interacted directly with MIR210HG promoters (**Figures 2F, G**). Taken together, these findings suggest that MIR210HG is a hypoxia-induced lncRNA in TNBC.

### MIR210HG Facilitates Aerobic Glycolysis in TNBC Cells

**Figure 3:**
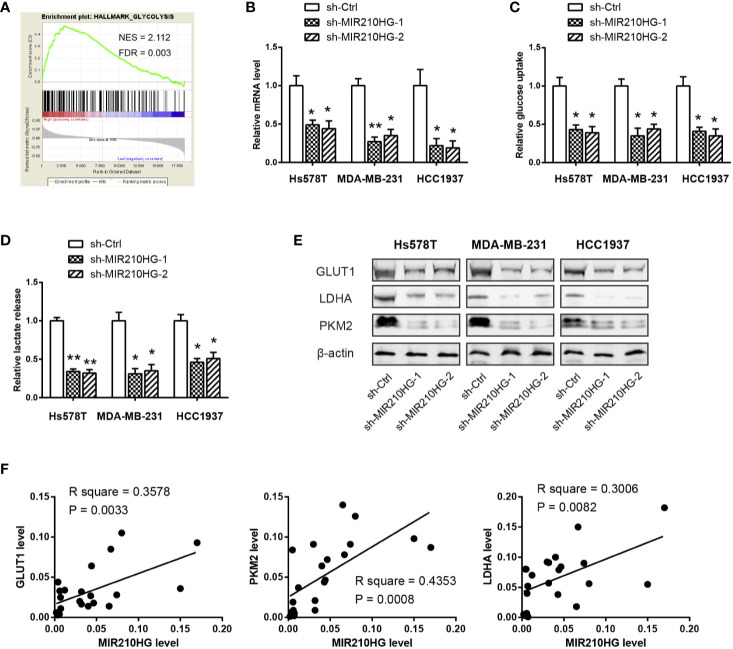
MIR210HG facilitates aerobic glycolysis in TNBC cells. **(A)** Gene set enrichment analysis (GSEA) showed that MIR210HG is closely related to glycolysis gene signature in TNBC. **(B)** Real-time qPCR analysis of MIR210HG knockdown efficiency in Hs578T, MDA-MB-231, and HCC1937 cells. **(C, D)** Quantification of glucose consumption and lactate production in Hs578T, MDA-MB-231, and HCC1937 cells after MIR210HG knockdown. **(E)** Western blotting analysis of the effect of MIR210HG knockdown on GLUT1, LDHA, and PKM2 expression in Hs578T, MDA-MB-231, and HCC1937 cells. **(F)** Correlation analysis of MIR210HG and three glycolytic components in 22 TNBC tissues. *p < 0.05; **p < 0.01.

To elucidate the cellular functions of MIR210HG in TNBC, we compared the gene expression profiles between MIR210HG-low group and MIR210HG-high group by using the RNA-seq data in the TCGA cohort. Interestingly, gene set enrichment analysis (GSEA) showed that glycolysis gene signature was significantly enriched in samples with high MIR210HG expression (**Figure 3A**). To further confirm this observation, we generated stably sh-MIR210HG expressing cell lines (**Figure 3B**). MIR210HG knockdown led to marked reduction in glucose consumption and lactate production in Hs578T, MDA-MB-231 and HCC1937 cells (**Figures 3C, D**). Western blotting analysis showed that MIR210HG knockdown attenuated the protein expression of three key glycolytic components (GLUT1, PKM2 and LDHA) (**Figure 3E**). Moreover, real-time qPCR analysis revealed that MIR210HG expression was closely associated with GLUT1, PKM2 and LDHA mRNA expression in 22 TNBC tissues (**Figure 3F**). Collectively, MIR210HG may act as a positive glycolysis modulator in TNBC.

### MIR210HG Promotes HIF1α Translation in TNBC

**Figure 4:**
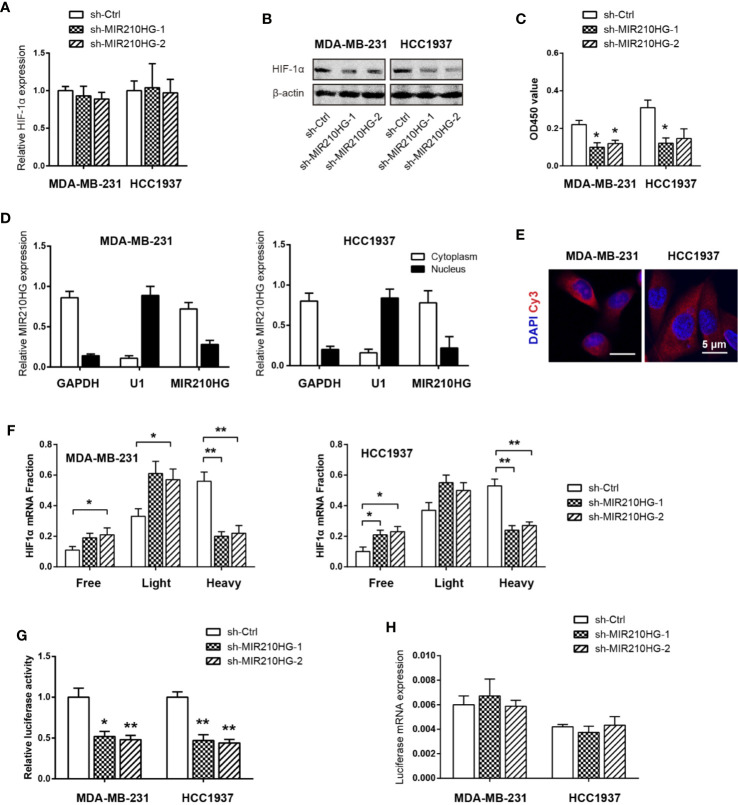
MIR210HG upregulates HIF1α translation in TNBC. **(A)** Real-time qPCR analysis of HIF-1α mRNA expression in MDA-MB-231 and HCC1937 cells stably expressing sh-MIR210HG or sh-Ctrl. **(B)** Western blotting analysis of HIF-1α protein expression in MDA-MB-231, and HCC1937 cells stably expressing sh-MIR210HG or sh-Ctrl. **(C)** Effects of MIR210HG knockdown on the HIF-1α transcriptional activity. **(D)** Real-time qPCR analysis of HIF-1α location in MDA-MB-231 and HCC1937 cells. GAPDH and U1 were used as internal cytoplasmic and nuclear control, respectively. **(E)** FISH analysis of HIF-1α location in MDA-MB-231 and HCC1937 cells. Scale bar: 5 μm. **(F)** Relative distribution of HIF-1α mRNA across the polysome fractions in cells stably expressing sh-MIR210HG or sh-Ctrl. **(G)** Effect of MIR210HG knockdown on the translation activity of 5’-UTR of HIF-1α mRNA in MDA-MB-231 and HCC1937 cells was detected by luciferase reporter assay. **(H)** Real-time qPCR analysis of the luciferase transcript expression in MDA-MB-231 and HCC1937 cells. *p < 0.05; **p < 0.01.

In light of the critical role of HIF-1α in regulating the Warburg effect (32), we tested whether MIR210HG facilitates aerobic glycolysis via HIF-1α. Real-time qPCR analysis showed that no significant change in HIF-1α mRNA levels in response to MIR210HG knockdown (**Figure 4A**). In contrast with this observation, western blotting result showed that MIR210HG knockdown led to marked reduction in HIF-1α protein expression (**Figure 4B**), suggesting that the regulation of HIF-1α by MIR210HG is post-transcriptional. Consistently, HIF-1α transcriptional activity was also downregulated by MIR210HG knockdown (**Figure 4C**). To uncover the mechanism by which MIR210HG promotes HIF-1α mRNA translation, we detected the subcellular localization of MIR210HG in MDA-MB-231 and HCC1937 cells. The result showed that MIR210HG was predominantly located in the cytoplasm, which was consistent with its potential role in post-transcriptional regulation of HIF-1α (**Figure 4D**). Fluorescence in situ hybridization of MIR210HG in MDA-MB-231 and HCC1937 cells also confirmed the cytoplasm localization (**Figure 4E**). Next, we characterized polysome-associated HIF-1α mRNA in MIR210HG knockdown cells. As a result, MIR210HG knockdown increased association of HIF-1α to free and light ribosome fractions and decreased association of HIF-1α mRNA to heavy ribosome fractions (**Figure 4F**), indicating that MIR210HG regulates HIF-1α translation. To determine whether MIR210HG regulates HIF-1α translation through binding its 5’-UTR, we generated a bicistronic reporter containing HIF-1α 5’-UTR and performed luciferase reporter assay. Indeed, the luciferase reporter activity was significantly reduced in sh-MIR210HG MDA-MB-231 and HCC1937 cells compared with control cells (**Figure 4G**) whereas the mRNA levels of luciferase were not changed (**Figure 4H**).

### MIR210HG Knockdown Suppresses Tumor Growth

**Figure 5:**
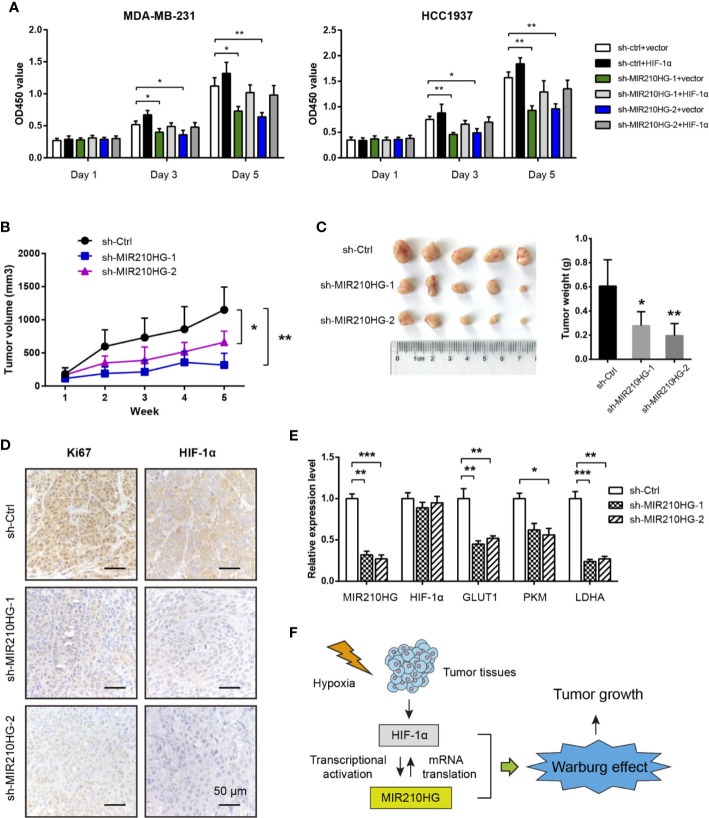
MIR210HG knockdown suppresses tumor growth. **(A)** CCK-8 analysis of the effect of MIR210HG knockdown in the cell proliferation of MDA-MB-231 and HCC1937 cells with or without HIF-1α expression. **(B)** The curve of tumor volume in the indicated three groups (sh-Ctrl, sh-MIR210HG-1, and sh-MIR210HG-1). **(C)** The tumor weights in the indicated three groups (sh-Ctrl, sh-MIR210HG-1, and sh-MIR210HG-1). **(D)** IHC analysis of Ki67 and HIF-1α protein in xenograft tumor tissues. Scale bar: 50 μm. **(E)** Real-time qPCR analysis of MIR210HG, HIF-1α, GLUT1, PKM2, and LDHA in xenograft tumor tissues. **(F)** Proposed model illustrating the mechanism by which HIF-1α-MIR210HG feedback loop promotes the Warburg effect and facilitate tumor growth in TNBC. *p < 0.05; **p < 0.01; ***p < 0.001.

By CCK-8 experiment, we showed that MIR210HG knockdown significantly inhibited cell proliferation of MDA-MB-231 and HCC1937 cells (**Figure 5A**). Moreover, reduced cell viability induced by MIR210HG knockdown can be largely restored by ectopic expression of HIF-1α (**Figure 5A**). To test the *in vivo* oncogenic role of MIR210HG in TNBC, we established subcutaneous xenograft tumor model by injection of sh-Ctrl or sh-MIR210HG MDA-MB-231 cells into nude mice (n = 5 per group). Tumor volume was monitored for 5 consecutive weeks. As shown in **Figure 5B**, MIR210HG knockdown significantly retarded tumor growth. Five weeks later, mice were sacrificed and tumors were weighted. MIR210HG knockdown drastically reduced tumor weight (**Figure 5C**). IHC analysis showed that Ki67 and HIF-1α positive staining were also decreased in sh-MIR210HG tumor tissues compared with that in sh-Ctrl group (**Figure 5D**). Consistently, real-time qPCR analysis showed that MIR210HG knockdown did not affect HIF-1α mRNA but reduced the expression of GLUT1, PKM2 and LDHA (**Figure 5E**). Taken together, these findings suggest that a positive feedback loop between MIR210HG and HIF-1α may enhance Warburg effect, which ultimately promotes tumor growth in TNBC (**Figure 5F**).

### MIR210HG Is Highly Expressed in Breast Cancer and Predicts a Poor Prognosis

**Figure 6:**
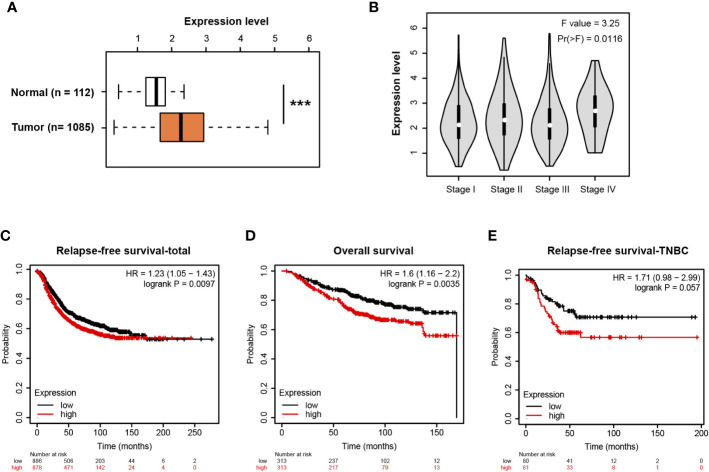
MIR210HG is highly expressed in breast cancer and predicts a poor prognosis. **(A)** MIR210HG expression in breast cancer and normal control tissues; data was acquired from TCGA + GTEx cohort. **(B)** MIR210HG expression in different stages of breast cancer tissues. **(C)** Kaplan-Meier survival curves showing an association between MIR210HG expression and relapse-free survival in patients with breast cancer. **(D)** Kaplan-Meier survival curves showing an association between MIR210HG expression and overall survival in patients with breast cancer. **(E)** Kaplan-Meier survival curves showing an association between MIR210HG expression and relapse-free survival in patients with TNBC. ***: p < 0.001.

Using the transcriptomic profiles from TCGA and the Genotype-Tissue Expression (GTEx) portal, we analyzed MIR210HG in breast cancer tissues and normal breast tissues. The result showed that MIR210HG expression was significantly upregulated in breast tumor tissues compared with normal controls (**Figure 6A**) and there was an increase in stage IV tumors (**Figure 6B**). By Kaplan-Meier plotter analysis (https://kmplot.com/analysis/) (33), we found that MIR210HG expression predicted a poor prognosis in relapse-free survival (**Figure 6C**) and overall survival (**Figure 6D**) in breast cancer patients. Importantly, higher MIR210HG expression showed a more significant hazard ratio for relapse-free survival in TNBC patients (**Figure 6E**).

## Discussion

Recently, lncRNAs are emerged as important regulators of gene expression at chromatin, transcriptional and posttranscriptional levels with diverse functions in many physiological and pathological processes, especially cancer. Hypoxia is a typical feature of tumor microenvironment and is essential for aggressive cancer phenotypes. Under hypoxia, HIF-1α stimulates expression of multiple hypoxia responsive genes via binding to the hypoxia response elements (HREs), eliciting a wide spectrum of cellular adaptations, such as angiogenesis, proliferation, and metabolic reprogramming. Not surprisingly, lncRNAs are also downstream targets of HIF-1α and act as effectors in response to hypoxia. In this study, we identified two dysregulated hypoxia-related lncRNAs (XIST and MIR210HG) in TNBC. Many studies have documented the tumor suppressor function of XIST in breast cancer and TNBC, which is consistent with its expression pattern as revealed in this study (34–36). From the therapeutic point of view, we focused on the study of the role and mechanism of MIR210HG in TNBC.

MIR210HG is the host gene of miR-210 and is well-known hypoxia lncRNA induced by HIF-1α.

In varicocele-related male infertility, MIR210HG was identified as a hypoxia-related long noncoding RNAs (37). Similarly, MIR210HG expression was also induced in hypoxic human umbilical vein endothelial cells (HUVECs) (38) and human proximal tubular epithelial cells (PTECs) (39). Consistent with these reports, we for the first time revealed that MIR210HG was transcriptionally activated by in HIF-1α under hypoxic conditions. Previously, MIR210HG has been demonstrated to be overexpressed in hepatocellular carcinoma (40), non-small cell lung cancer (NSCLC) (41), osteosarcoma (42), glioma (43) and chemoresistant pancreatic cancer (44). In invasive breast cancer patients, MIR210HG is highly expressed and confers a poor prognosis (45). Here, we confirmed this finding and showed that MIR210HG was closely associated with a relapse-free survival in TNBC. The high expression of MIR210HG in TNBC is associated with poor prognosis, suggesting that MIR210HG may be used as a potential prognostic predictor. Moreover, we revealed that MIR210HG was highly expressed in TNBC cells in comparison to the non-TNBC cells and the nonmalignant MCF10A cells, suggesting a specific role of MIR210HG in TNBC development. However, the reason for differentially expressed MIR210HG in TNBC warrants further investigations. Given its expression pattern and prognostic value in TNBC, MIR210HG may represent a novel therapeutic target for TNBC treatment.

Several molecular mechanisms underlying the oncogenic roles of MIR210HG have been reported. In cervical cancer, MIR210HG might act as a competing endogenous RNA (ceRNA) of miR-503-5p to relieve the suppressive effect of miR-503-5p on TRAF4 expression, resulting in increased cell proliferation and invasive capacity (46). In NSCLC, MIR210HG is able to promote cell proliferation and invasion through targeting miR-874/STAT3 axis and regulating methylation of CACNA2D2 promoter via binding to DNMT1 (41, 47). In this study, we identified a novel function of MIR210HG in regulating the Warburg effect. In line with our result, RUAN et al. showed that higher MIR210HG expression was associated with shorter overall survival in colon cancer and MIR210HG may play a role in the modulation of energy metabolism, especially glucose metabolism (48). Polysome profiling of HIF-1α mRNA showed reduced translation of HIF-1α in MIR210HG knockdown cells, suggesting the regulatory role if MIR210HG in HIF-1a translation process. Thus, we provided a previous unprecedented mechanism by which MIR210HG promotes tumor progression. Our study advances the knowledge of the regulation of HIF-1α, and underlines the essential relevance of lncRNA in gene regulation. Although our data indicated that MIR210HG exerts a regulatory role in HIF-1α, the underlying mechanisms of MIR210HG in interacting with HIF-1α remained to be determined in following studies. Additionally, MIR210HG can act as a ceRNA of miR-1226-3p to regulate mucin-1c expression resulting in increased breast cancer metastasis (45). Therefore, we cannot exclude other alternative targets and potential cellular mechanisms of MIR210HG in regulating the glycolytic phenotype of TNBC.

### Conclusion

To the best of our knowledge, the present study provides the first evidence that MIR210HG acts as a metabolic regulator to promote TNBC cell proliferation and tumor growth. Our study revealed that hypoxia-induced MIR210HG might act as a tumor promoter by enhancing the Warburg effect. Molecular mechanism showed that MIR210HG regulates the expression of HIF-1α at the translational level. Our findings shed lights on the HIF-1α/MIR210HG feedback loop in TNBC glucose metabolism and suggest that targeting HIF-1α/MIR210HG axis might serve as new strategies for TNBC prevention and therapy.

## Data Availability Statement

All datasets presented in this study are included in the article/**Supplementary Material**.

## Ethics Statement

The studies involving human participants were reviewed and approved by The First Hospital of Jilin University. The patients/participants provided their written informed consent to participate in this study. The animal study was reviewed and approved by The First Hospital of Jilin University.

## Author Contributions

DS and S-HJ designed and performed the research. YD, NW, and RM performed the experiments. DS, YD, and NW analyzed and interpreted data, and wrote the draft manuscript. All authors contributed to the writing and reviewing of the manuscript. All authors contributed to the article and approved the submitted version.

## Funding

This work was supported by grants from National Natural Science Foundation of China (81773171) and Science and Technology Department of Jilin Province (20170311005YY, 20200404197YY, and 20200201349JC).

## Conflict of Interest

The authors declare that the research was conducted in the absence of any commercial or financial relationships that could be construed as a potential conflict of interest.

## Abbreviations

TNBC, triple-negative breast cancer; lncRNA, long noncoding RNA; HER2, human epidermal growth factor receptor 2; HIF-1α, hypoxia-inducible factor 1 alpha; TCGA, The Cancer Genome Atlas; IHC, Immunohistochemistry; co-IP, chromatin immunoprecipitation; GTEx, Genotype-Tissue Expression; HUVECs, human umbilical vein endothelial cells; PTECs, human proximal tubular epithelial cells; ceRNA, competing endogenous RNA.

## References

[1] Bianchini G Balko JM Mayer IA Sanders ME Gianni L Triple-negative breast cancer: challenges and opportunities of a heterogeneous disease. Nat Rev Clin Oncol (2016) 13:674–90. 10.1038/nrclinonc.2016.66 PMC5461122 27184417

[2] Diana A Carlino F Franzese E Oikonomidou O Criscitiello C De Vita F Early Triple Negative Breast Cancer: Conventional Treatment and Emerging Therapeutic Landscapes. Cancers (Basel) (2020) 12:819. 10.3390/cancers12040819 PMC7225917 32235297

[3] Zhao S Zuo WJ Shao ZM Jiang YZ Molecular subtypes and precision treatment of triple-negative breast cancer. Ann Transl Med (2020) 8:499. 10.21037/atm.2020.03.194 32395543 PMC7210152

[4] Bertout JA Patel SA Simon MC The impact of O2 availability on human cancer. Nat Rev Cancer (2008) 8:967–75. 10.1038/nrc2540 PMC3140692 18987634

[5] Wilson WR Hay MP Targeting hypoxia in cancer therapy. Nat Rev Cancer (2011) 11:393–410. 10.1038/nrc3064 21606941

[6] Kung-Chun Chiu D Pui-Wah Tse A Law CT Ming-Jing Xu I Lee D Chen M Hypoxia regulates the mitochondrial activity of hepatocellular carcinoma cells through HIF/HEY1/PINK1 pathway. Cell Death Dis (2019) 10:934. 10.1038/s41419-019-2155-3 31819034 PMC6901483

[7] Ling S Shan Q Zhan Q Ye Q Liu P Xu S USP22 promotes hypoxia-induced hepatocellular carcinoma stemness by a HIF1alpha/USP22 positive feedback loop upon TP53 inactivation. Gut (2020) 69:1322–34. 10.1136/gutjnl-2019-319616 31776228

[8] Qureshi-Baig K Kuhn D Viry E Pozdeev VI Schmitz M Rodriguez F Hypoxia-induced autophagy drives colorectal cancer initiation and progression by activating the PRKC/PKC-EZR (ezrin) pathway. Autophagy (2019) 16:1436–52. 10.1080/15548627.2019.1687213 PMC7469473 31775562

[9] Jing X Yang F Shao C Wei K Xie M Shen H Role of hypoxia in cancer therapy by regulating the tumor microenvironment. Mol Cancer (2019) 18:157. 10.1186/s12943-019-1089-9 31711497 PMC6844052

[10] Semenza GL The hypoxic tumor microenvironment: A driving force for breast cancer progression. Biochim Biophys Acta (2016) 1863:382–91. 10.1016/j.bbamcr.2015.05.036 PMC4678039 26079100

[11] Ye Y Hu Q Chen H Liang K Yuan Y Xiang Y Characterization of Hypoxia-associated Molecular Features to Aid Hypoxia-Targeted Therapy. Nat Metab (2019) 1:431–44. 10.1038/s42255-019-0045-8 PMC6980239 31984309

[12] Moyer MW Targeting hypoxia brings breath of fresh air to cancer therapy. Nat Med (2012) 18:636–7. 10.1038/nm0512-636b 22561804

[13] Ulitsky I Bartel DP lincRNAs: genomics, evolution, and mechanisms. Cell (2013) 154:26–46. 10.1016/j.cell.2013.06.020 23827673 PMC3924787

[14] Hu WL Jin L Xu A Wang YF Thorne RF Zhang XD GUARDIN is a p53-responsive long non-coding RNA that is essential for genomic stability. Nat Cell Biol (2018) 20:492–502. 10.1038/s41556-018-0066-7 29593331

[15] Liu K Gao L Ma X Huang JJ Chen J Zeng L Long non-coding RNAs regulate drug resistance in cancer. Mol Cancer (2020) 19:54. 10.1186/s12943-020-01162-0 32164712 PMC7066752

[16] Jin KT Lu ZB Lv JQ Zhang JG The role of long non-coding RNAs in mediating chemoresistance by modulating autophagy in cancer. RNA Biol (2020) 17:1727–40. 10.1080/15476286.2020.1737787 PMC7714480 32129701

[17] Zheng S Yang L Zou Y Liang JY Liu P Gao G Long non-coding RNA HUMT hypomethylation promotes lymphangiogenesis and metastasis via activating FOXK1 transcription in triple-negative breast cancer. J Hematol Oncol (2020) 13:17. 10.1186/s13045-020-00861-x 32138762 PMC7059688

[18] Zhang H Zhang N Liu Y Su P Liang Y Li Y Epigenetic Regulation of NAMPT by NAMPT-AS Drives Metastatic Progression in Triple-Negative Breast Cancer. Cancer Res (2019) 79:3347–59. 10.1158/0008-5472.CAN-18-3418 30940661

[19] Shin VY Chen J Cheuk IW Siu MT Ho CW Wang X Long non-coding RNA NEAT1 confers oncogenic role in triple-negative breast cancer through modulating chemoresistance and cancer stemness. Cell Death Dis (2019) 10:270. 10.1038/s41419-019-1513-5 30894512 PMC6426882

[20] Gooding AJ Zhang B Gunawardane L Beard A Valadkhan S Schiemann WP The lncRNA BORG facilitates the survival and chemoresistance of triple-negative breast cancers. Oncogene (2019) 38:2020–41. 10.1038/s41388-018-0586-4 PMC6430670 30467380

[21] Hu Q Ye Y Chan LC Li Y Liang K Lin A Oncogenic lncRNA downregulates cancer cell antigen presentation and intrinsic tumor suppression. Nat Immunol (2019) 20:835–51. 10.1038/s41590-019-0400-7 PMC6619502 31160797

[22] Vidovic D Huynh TT Konda P Dean C Cruickshank BM Sultan M ALDH1A3-regulated long non-coding RNA NRAD1 is a potential novel target for triple-negative breast tumors and cancer stem cells. Cell Death Differ (2020) 27:363–78. 10.1038/s41418-019-0362-1 PMC7206030 31197235

[23] Hanahan D Weinberg RA Hallmarks of cancer: the next generation. Cell (2011) 144:646–74. 10.1016/j.cell.2011.02.013 21376230

[24] Zhao Y Butler EB Tan M Targeting cellular metabolism to improve cancer therapeutics. Cell Death Dis (2013) 4:e532. 10.1038/cddis.2013.60 23470539 PMC3613838

[25] Vander Heiden MG Cantley LC Thompson CB Understanding the Warburg effect: the metabolic requirements of cell proliferation. Science (2009) 324:1029–33. 10.1126/science.1160809 PMC2849637 19460998

[26] Zhang J Yang J Lin C Liu W Huo Y Yang M Endoplasmic Reticulum stress-dependent expression of ERO1L promotes aerobic glycolysis in Pancreatic Cancer. Theranostics (2020) 10:8400–14. 10.7150/thno.45124 PMC7381747 32724477

[27] Iansante V Choy PM Fung SW Liu Y Chai JG Dyson J PARP14 promotes the Warburg effect in hepatocellular carcinoma by inhibiting JNK1-dependent PKM2 phosphorylation and activation. Nat Commun (2015) 6:7882. 10.1038/ncomms8882 26258887 PMC4918319

[28] Buffa FM Harris AL West CM Miller CJ Large meta-analysis of multiple cancers reveals a common, compact and highly prognostic hypoxia metagene. Br J Cancer (2010) 102:428–35. 10.1038/sj.bjc.6605450 PMC2816644 20087356

[29] Jiang SH Li J Dong FY Yang JY Liu DJ Yang XM Increased Serotonin Signaling Contributes to the Warburg Effect in Pancreatic Tumor Cells Under Metabolic Stress and Promotes Growth of Pancreatic Tumors in Mice. Gastroenterology (2017) 153:277–91 e219. 10.1053/j.gastro.2017.03.008 28315323

[30] Malakar P Stein I Saragovi A Winkler R Stern-Ginossar N Berger M Long Noncoding RNA MALAT1 Regulates Cancer Glucose Metabolism by Enhancing mTOR-Mediated Translation of TCF7L2. Cancer Res (2019) 79:2480–93. 10.1158/0008-5472.CAN-18-1432 30914432

[31] Li R Wang Y Zhang X Feng M Ma J Li J Exosome-mediated secretion of LOXL4 promotes hepatocellular carcinoma cell invasion and metastasis. Mol Cancer (2019) 18:18. 10.1186/s12943-019-0948-8 30704479 PMC6354392

[32] Cairns RA Harris IS Mak TW Regulation of cancer cell metabolism. Nat Rev Cancer (2011) 11:85–95. 10.1038/nrc2981 21258394

[33] Gyorffy B Lanczky A Eklund AC Denkert C Budczies J Li Q An online survival analysis tool to rapidly assess the effect of 22,277 genes on breast cancer prognosis using microarray data of 1,809 patients. Breast Cancer Res Treat (2010) 123:725–31. 10.1007/s10549-009-0674-9 20020197

[34] Li X Hou L Yin L Zhao S LncRNA XIST interacts with miR-454 to inhibit cells proliferation, epithelial mesenchymal transition and induces apoptosis in triple-negative breast cancer. J Biosci (2020) 45:45. 10.1007/s12038-020-9999-7 32098924

[35] Xing F Liu Y Wu SY Wu K Sharma S Mo YY Loss of XIST in Breast Cancer Activates MSN-c-Met and Reprograms Microglia via Exosomal miRNA to Promote Brain Metastasis. Cancer Res (2018) 78:4316–30. 10.1158/0008-5472.CAN-18-1102 PMC6072593 30026327

[36] Zheng R Lin S Guan L Yuan H Liu K Liu C Long non-coding RNA XIST inhibited breast cancer cell growth, migration, and invasion via miR-155/CDX1 axis. Biochem Biophys Res Commun (2018) 498:1002–8. 10.1016/j.bbrc.2018.03.104 29550489

[37] Ata-Abadi NS Mowla SJ Aboutalebi F Dormiani K Kiani-Esfahani A Tavalaee M Hypoxia-related long noncoding RNAs are associated with varicocele-related male infertility. PloS One (2020) 15:e0232357. 10.1371/journal.pone.0232357 32353040 PMC7192471

[38] Voellenkle C Garcia-Manteiga JM Pedrotti S Perfetti A De Toma I Da Silva D Implication of Long noncoding RNAs in the endothelial cell response to hypoxia revealed by RNA-sequencing. Sci Rep (2016) 6:24141. 10.1038/srep24141 27063004 PMC4827084

[39] Lin J Zhang X Xue C Zhang H Shashaty MG Gosai SJ The long noncoding RNA landscape in hypoxic and inflammatory renal epithelial injury. Am J Physiol Renal Physiol (2015) 309:F901–13. 10.1152/ajprenal.00290.2015 PMC4669357 26400545

[40] Wang Y Li W Chen X Li Y Wen P Xu F MIR210HG predicts poor prognosis and functions as an oncogenic lncRNA in hepatocellular carcinoma. BioMed Pharmacother (2019) 111:1297–301. 10.1016/j.biopha.2018.12.134 30841443

[41] Kang X Kong F Huang K Li L Li Z Wang X LncRNA MIR210HG promotes proliferation and invasion of non-small cell lung cancer by upregulating methylation of CACNA2D2 promoter via binding to DNMT1. Onco Targets Ther (2019) 12:3779–90. 10.2147/OTT.S189468 PMC6529604 31190878

[42] Li J Wu QM Wang XQ Zhang CQ Long Noncoding RNA miR210HG Sponges miR-503 to Facilitate Osteosarcoma Cell Invasion and Metastasis. DNA Cell Biol (2017) 36:1117–25. 10.1089/dna.2017.3888 28972855

[43] Min W Dai D Wang J Zhang D Zhang Y Han G Long Noncoding RNA miR210HG as a Potential Biomarker for the Diagnosis of Glioma. PloS One (2016) 11:e0160451. 10.1371/journal.pone.0160451 27673330 PMC5038942

[44] Li D Qian X Xu P Wang X Li Z Qian J Identification of lncRNAs and Their Functional Network Associated with Chemoresistance in SW1990/GZ Pancreatic Cancer Cells by RNA Sequencing. DNA Cell Biol (2018) 37:839–49. 10.1089/dna.2018.4312 30113217

[45] Li XY Zhou LY Luo H Zhu Q Zuo L Liu GY The long noncoding RNA MIR210HG promotes tumor metastasis by acting as a ceRNA of miR-1226-3p to regulate mucin-1c expression in invasive breast cancer. Aging (Albany NY) (2019) 11:5646–65. 10.18632/aging.102149 PMC6710038 31399552

[46] Wang AH Jin CH Cui GY Li HY Wang Y Yu JJ MIR210HG promotes cell proliferation and invasion by regulating miR-503-5p/TRAF4 axis in cervical cancer. Aging (Albany NY) (2020) 12:3205–17. 10.18632/aging.102799 PMC7066889 32087604

[47] Bu L Zhang L Tian M Zheng Z Tang H Yang Q LncRNA MIR210HG Facilitates Non-Small Cell Lung Cancer Progression Through Directly Regulation of miR-874/STAT3 Axis. Dose Response (2020) 18:1559325820918052. 10.1177/1559325820918052 32699535 PMC7357071

[48] Ruan Z Xu Z Li Z Lv Y Integral analyses of survival-related long non-coding RNA MIR210HG and its prognostic role in colon cancer. Oncol Lett (2019) 18:1107–16. 10.3892/ol.2019.10435 PMC6607050 31423171

