# Supplementary material for: Long Noncoding RNA MIR210HG Promotes the Warburg Effect and Tumor Growth by Enhancing HIF-1α Translation in Triple-Negative Breast Cancer
Source: Front Oncol. 2020 Dec 17;10:580176. doi: 10.3389/fonc.2020.580176 (PMC7774020; doi:10.3389/fonc.2020.580176)
Supplement: Supplementary file 1 [file DataSheet_1.pdf]

Supplementary Material

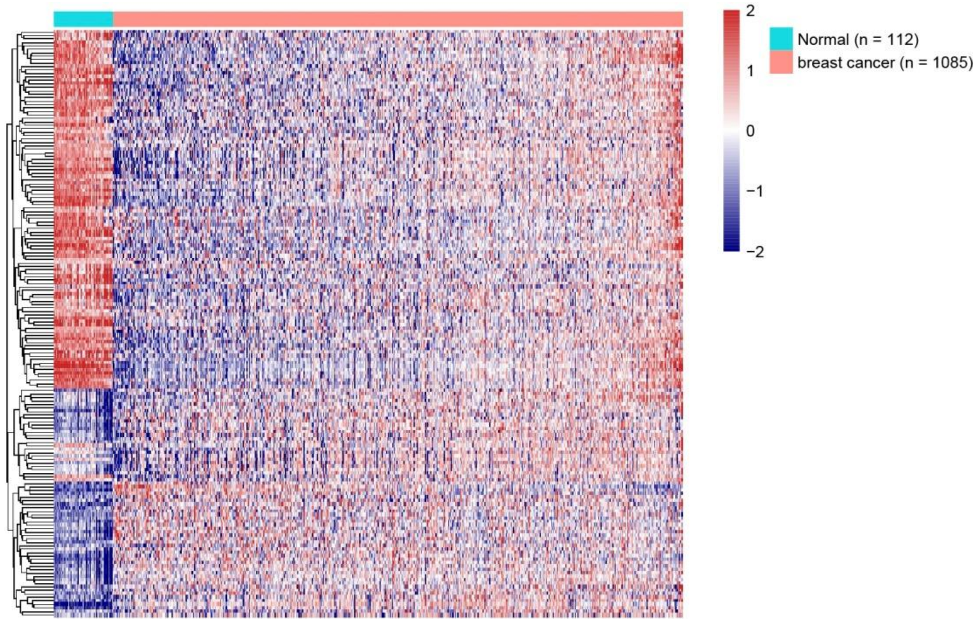

Supplementary Figure 1. Heatmap of differentially expressed lncRNAs in breast tumor tissues (n= 1085) compared with corresponding normal tissues (n = 112).

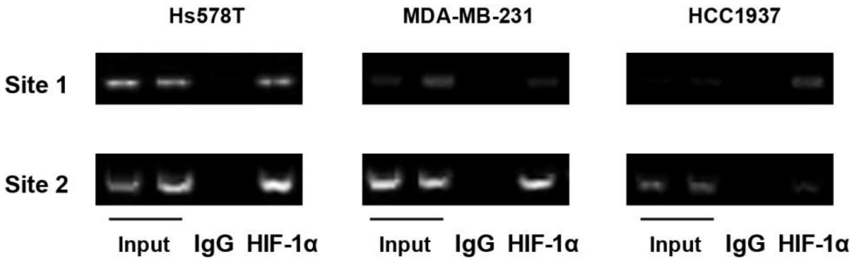

Supplementary Figure 2. The CHIP-PCR products in each group as indicated.

Supplementary table 1 The sequences of primers used in this study

Gene	Forward primer (5'-3')	Reverse primer (5'-3')
MIR21	GCTTGGTAGAGTGTCACGCC	CATCTGACCGAGCCAGTTTG
HIF-1α	GAACGTCGAAAAGAAAAGTCTCG	CCTTATCAAGATGCGAACTCACA
GLUT1	ATTGGCTCCGGTATCGTCAAC	GCTCAGATAGGACATCCAGGGTA
PKM2	ATAACGCCTACATGGAAAAGTGT	TAAGCCCATCATCCACGTAGA
LDHA	ATGGCAACTCTAAAGGATCAGC	CCAACCCCAACAACTGTAATCT
ACTB	CATGTACGTTGCTATCCAGGC	CTCCTTAATGTCACGCACGAT

Supplementary table 2 Detailed clinical information of all TNBC patients enrolled in this study

Sample ID	Stage	Age	Gender	Metastasis status	Node status	OS_Time	OS_event	XIST	MIR210HG	hypoxia score	Group
TCGA-A1-A0SK-01	Stage IIA	54	FEMALE	M0	N0	967	1	7.542	2.101	0.310	H
TCGA-A1-A0SP-01	Stage IIA	40	FEMALE	M0	N0	583	0	2.831	2.461	0.027	H
TCGA-A2-A04U-01	Stage IIA	47	FEMALE	M0	N0	670	0	3.831	0.411	-0.098	L
TCGA-A2-A0CM-01	Stage IIA	40	FEMALE	M0	N0	754	1	5.503	-0.593	-0.168	L
TCGA-A2-A0D0-01	Stage IIA	60	FEMALE	M0	N0	643	0	0.098	1.529	0.705	H
TCGA-A2-A0D2-01	Stage IIB	45	FEMALE	M0	N0	1027	0	4.452	0.729	0.138	H
TCGA-A2-A0SX-01	Stage IA	48	FEMALE	M0	N0	1288	0	6.979	1.466	-0.648	L
TCGA-A2-A0T0-01	Stage IIB	59	FEMALE	M0	N1	533	0	2.814	3.259	0.341	H
TCGA-A2-A0T2-01	Stage IV	66	FEMALE	M1	N3	240	1	2.426	4.544	0.447	H
TCGA-A2-A0YE-01	Stage IIB	48	FEMALE	M0	N1	553	0	3.674	2.356	-0.124	L
TCGA-A2-A1G6-01	Stage IIIA	50	FEMALE	M0	N2	132	0	5.589	-0.207	-0.758	L
TCGA-A2-A3XT-01		NA				NA	NA	6.149	0.611	-0.008	H
TCGA-A2-A3XX-01		NA				NA	NA	2.487	1.724	0.314	H
TCGA-A2-A3XY-01		NA				NA	NA	6.190	1.406	-0.027	H
TCGA-A7-A0DA-01	Stage IIA	62	FEMALE	M0	N0	373	0	5.584	2.788	-0.077	L
TCGA-A7-A26G-01	Stage IIA	50	FEMALE	M0	N0	209	0	7.261	-1.156	-0.621	L
TCGA-A7-A4SE-01		NA				NA	NA	6.790	3.661	0.309	H
TCGA-A7-A6VV-01		NA				NA	NA	5.031	2.829	0.688	H
TCGA-A7-A6VW-01		NA				NA	NA	0.012	2.799	0.489	H
TCGA-A7-A6VY-01		NA				NA	NA	2.736	3.680	0.637	H
TCGA-A8-A07C-01	Stage IIA	57	FEMALE	M0	N0	580	0	4.706	0.068	-0.590	L
TCGA-A8-A07O-01	Stage IIA	51	FEMALE	M0	N0	304	0	3.840	2.205	0.626	H
TCGA-A8-A08R-01	Stage IIB	52	FEMALE	M0	N1	30	0	5.490	0.808	-0.385	L

TCGA-A8-A09X-01	Stage IIIC	62	FEMALE	M0	N3	426	1	6.664	-1.151	-0.224	L
TCGA-AC-A2BK-01		NA				NA	NA	2.349	-0.568	-0.092	L
TCGA-AC-A2QH-01		NA				NA	NA	12.688	2.337	-0.571	L
TCGA-AC-A2QJ-01		NA				NA	NA	1.077	4.406	0.306	H
TCGA-AC-A6IW-01		NA				NA	NA	2.692	-2.272	-0.082	L
TCGA-AC-A7VC-01		NA				NA	NA	3.901	2.223	0.480	H
TCGA-AN-A04D-01	Stage IIB	58	FEMALE	M0	N1	52	0	5.879	1.739	-0.577	L
TCGA-AN-A0AL-01	Stage IIIB	41	FEMALE	M0	N0	197	0	4.643	1.789	0.194	H
TCGA-AN-A0AR-01	Stage IIA	55	FEMALE	M0	N0	9	0	4.962	1.750	-0.456	L
TCGA-AN-A0AT-01	Stage IIA	62	FEMALE	M0	N0	10	0	5.368	2.439	0.611	H
TCGA-AN-A0G0-01	Stage IIA	56	FEMALE	M0	N0	16	0	4.198	2.433	0.051	H
TCGA-AN-A0XU-01	Stage IIA	54	FEMALE	M0	N0	10	0	4.205	2.055	0.330	H
TCGA-AO-A03U-01	Stage IA	31	FEMALE	M0	N0	1796	1	7.158	1.323	-0.316	L
TCGA-AO-A0J4-01	Stage IA	41	FEMALE	M0	N0	294	0	4.805	2.400	0.332	H
TCGA-AO-A0J6-01	Stage IIA	61	FEMALE	M0	N0	775	0	1.238	3.814	0.450	H
TCGA-AO-A0JL-01	Stage IIIA	59	FEMALE	M0	N2	1319	0	5.458	2.704	-0.490	L
TCGA-AO-A124-01	Stage IIA	38	FEMALE	M0	N0	3119	0	2.374	1.733	0.226	H
TCGA-AO-A128-01	Stage IIA	61	FEMALE	M0	N0	2877	0	7.198	1.324	-0.606	L
TCGA-AO-A129-01	Stage IIB	29	FEMALE	M0	N1	2923	0	5.180	3.119	0.175	H
TCGA-AO-A12F-01	Stage IIA	36	FEMALE	M0	N0	1471	0	5.335	0.052	-0.345	L
TCGA-AO-A1KR-01		51	FEMALE			2140	0	2.077	3.269	-0.169	L
TCGA-AQ-A04J-01	Stage IIA	45	FEMALE	M0	N0	499	0	6.760	1.724	-0.429	L
TCGA-AR-A0TS-01	Stage II	46	FEMALE	M0	N1	1138	0	6.715	0.128	-0.681	L
TCGA-AR-A0TU-01	Stage II	35	FEMALE	M0	N0	360	0	6.853	1.844	0.121	H
TCGA-AR-A0U4-01	Stage II	54	FEMALE	M0	N0	1627	0	7.070	2.799	0.322	H
TCGA-AR-A1AR-01	Stage III	50	FEMALE	M0	N2	523	1	6.924	-0.244	-0.359	L

TCGA-AR-A1AY-01	Stage I	65	FEMALE	M0	N0	614	0	4.723	1.319	0.341	H
TCGA-AR-A256-01	Stage II	45	FEMALE	M0	N0	2854	1	4.518	-0.894	-0.123	L
TCGA-AR-A2LR-01		NA				NA	NA	6.620	0.455	-0.500	L
TCGA-AR-A5QQ-01		NA				NA	NA	3.387	4.882	0.813	H
TCGA-B6-A3ZX-01		NA				NA	NA	6.915	1.609	-0.739	L
TCGA-B6-A400-01		NA				NA	NA	4.545	2.692	0.331	H
TCGA-B6-A402-01		NA				NA	NA	3.599	3.367	-0.386	L
TCGA-BH-A0B3-01	Stage IIB	53	FEMALE	M0	N1	1203	0	3.608	3.002	0.759	H
TCGA-BH-A0B9-01	Stage IA	44	FEMALE	M0	N0	1572	0	3.721	2.646	0.167	H
TCGA-BH-A0BG-01	Stage I	73	FEMALE	M0	N0	756	0	3.366	1.751	-0.497	L
TCGA-BH-A0BL-01	Stage I	35	FEMALE	M0	N0	1339	0	6.044	2.050	0.109	H
TCGA-BH-A0E0-01	Stage IIIC	38	FEMALE	M0	N3	133	0	6.256	3.565	0.116	H
TCGA-BH-A0RX-01	Stage IIA	59	FEMALE	M0	N0	170	0	4.553	0.794	-0.162	L
TCGA-BH-A0WA-01	Stage IA	82	FEMALE	M0	N0	372	0	7.925	2.824	0.734	H
TCGA-BH-A18G-01	Stage X	81	FEMALE	M0	N0	61	0	4.703	3.072	0.549	H
TCGA-BH-A18V-01	Stage IIB	48	FEMALE	M0	N1	1555	1	4.899	1.490	-0.217	L
TCGA-BH-A1EW-01	Stage IIA	38	FEMALE	M0	N1	1694	1	7.148	0.031	-0.505	L
TCGA-BH-A1F6-01		51	FEMALE			2965	1	3.042	1.971	0.297	H
TCGA-BH-A1FC-01	Stage IIA	78	FEMALE	M0	N1	3471	1	3.333	0.417	-0.394	L
TCGA-BH-A42U-01		NA				NA	NA	2.735	1.177	-0.588	L
TCGA-C8-A12V-01	Stage IIA	55	FEMALE	M0	N0	0	0	6.401	-1.520	-0.598	L
TCGA-C8-A131-01	Stage III	82	FEMALE	M0	N2	0	0	5.313	4.232	0.387	H
TCGA-C8-A1HJ-01	Stage II	53	FEMALE	M0	N0	5	0	9.198	-0.507	-0.544	L
TCGA-C8-A26X-01	Stage II	58	FEMALE	M0	N1	11	0	5.186	3.004	0.344	H
TCGA-C8-A26Y-01	Stage II	90	FEMALE	M0	N0	13	0	7.874	3.636	0.338	H
TCGA-C8-A27B-01	Stage IIB	48	FEMALE	M0	N0	30	0	3.995	2.240	0.166	H

TCGA-C8-A3M7-01		NA				NA	NA	7.683	0.002	-0.732	L
TCGA-D8-A13Z-01	Stage X	51	FEMALE	M0	N2	210	0	5.329	2.634	0.427	H
TCGA-D8-A143-01	Stage IIA	51	FEMALE	M0	N0	431	0	3.666	2.686	0.676	H
TCGA-D8-A147-01	Stage X	45	FEMALE	M0	N0	2	0	6.945	0.524	-0.555	L
TCGA-D8-A1JF-01	Stage IIIA	79	FEMALE	M0	N2	96	0	7.253	1.160	-0.568	L
TCGA-D8-A1JL-01	Stage IIA	72	FEMALE	M0	N0	265	0	5.980	1.097	-0.004	H
TCGA-D8-A1XK-01	Stage IB	55	FEMALE	M0	N1	326	0	5.759	0.839	0.655	H
TCGA-D8-A1XQ-01	Stage IIA	69	FEMALE	M0	N0	177	0	3.800	3.157	0.381	H
TCGA-D8-A27F-01	Stage IIA	40	FEMALE	M0	N0	227	0	4.309	2.057	0.409	H
TCGA-D8-A27H-01	Stage IIA	72	FEMALE	M0	N0	140	0	4.434	1.879	0.144	H
TCGA-D8-A27M-01	Stage IIA	59	FEMALE	M0	N0	144	0	7.914	2.797	-0.228	L
TCGA-E2-A14N-01	Stage IIB	37	FEMALE	M0	N1	1350	0	3.362	1.502	-0.022	H
TCGA-E2-A14R-01	Stage IIA	62	FEMALE	M0	N0	845	0	5.139	3.815	0.091	H
TCGA-E2-A14X-01	Stage IIIA	55	FEMALE	M0	N2	692	0	5.568	0.464	-0.613	L
TCGA-E2-A150-01	Stage IIA	48	FEMALE	M0	N0	591	0	4.675	0.624	0.379	H
TCGA-E2-A158-01	Stage IIA	43	FEMALE	M0	N1	450	0	7.840	0.772	-0.540	L
TCGA-E2-A1L7-01	Stage IIIA	40	FEMALE	M0	N2	633	0	8.224	-1.145	-0.349	L
TCGA-E2-A1LH-01	Stage I	59	FEMALE	M0	N0	2875	0	4.552	1.442	-0.296	L
TCGA-E2-A1LL-01	Stage IIIA	73	FEMALE	M0	N2	1014	0	3.074	1.362	0.780	H
TCGA-E2-A1LS-01	Stage IA	46	FEMALE	M0	N0	239	0	1.978	0.038	-0.691	L
TCGA-E9-A5FL-01		NA				NA	NA	0.921	4.844	0.355	H
TCGA-EW-A1OV-01	Stage IIB	56	FEMALE	M0	N1	522	0	6.732	0.113	-0.697	L
TCGA-EW-A1OW-01	Stage IIA	58	FEMALE	M0	N0	463	0	6.961	3.241	0.416	H
TCGA-EW-A1P4-01	Stage IIA	43	FEMALE	M0	N0	501	0	3.164	3.194	0.755	H
TCGA-EW-A1P8-01	Stage IIIC	58	FEMALE	M0	N3	239	1	1.722	2.093	0.101	H
TCGA-EW-A1PB-01	Stage IIIA	70	FEMALE	M0	N1	608	0	2.530	2.530	0.301	H

TCGA-EW-A1PH-01	Stage IIA	52	FEMALE	M0	N1	139	0	0.954	2.916	-0.027	L
TCGA-EW-A3U0-01		NA				NA	NA	6.187	0.091	-0.775	L
TCGA-EW-A6SB-01		NA				NA	NA	2.335	0.500	-0.585	L
TCGA-GI-A2C9-01		NA				NA	NA	6.888	0.222	-0.428	L
TCGA-GM-A2DB-01		62	FEMALE			1615	0	5.499	5.352	0.418	H
TCGA-GM-A2DF-01		53	FEMALE			1299	0	5.996	1.868	-0.560	L
TCGA-GM-A2DH-01		58	FEMALE			1286	0	7.029	1.820	-0.646	L
TCGA-HN-A2NL-01		NA				NA	NA	5.146	0.916	-0.533	L
TCGA-LL-A441-01		NA				NA	NA	6.501	0.251	-0.823	L
TCGA-LL-A5YO-01		NA				NA	NA	4.326	0.861	-0.630	L
TCGA-LL-A73Y-01		NA				NA	NA	5.493	1.860	-0.685	L
TCGA-OL-A6VO-01		NA				NA	NA	1.546	2.600	0.392	H
TCGA-S3-AA10-01		NA				NA	NA	4.379	0.975	-0.082	L
TCGA-S3-AA15-01		NA				NA	NA	6.175	1.002	-0.791	L
